# S-Adenosylmethionine: From the Discovery of Its Inhibition of Tumorigenesis to Its Use as a Therapeutic Agent

**DOI:** 10.3390/cells11030409

**Published:** 2022-01-25

**Authors:** Rosa M. Pascale, Maria M. Simile, Diego F. Calvisi, Claudio F. Feo, Francesco Feo

**Affiliations:** 1 Department of Medical, Surgical and Experimental Sciences, Division of Experimental Pathology and Oncology, University of Sassari, 07100 Sassari, Italy; simile@uniss.it (M.M.S.); calvisid@uniss.it (D.F.C.); feo@uniss.it (F.F.); 2 Department of Medical, Surgical and Experimental Sciences, Division of Surgery, University of Sassari, 07100 Sassari, Italy; cffeo@uniss.it

**Keywords:** S-adenosyl-L-methionine, methionine cycle, alcoholic liver disease, non-alcoholic fatty liver, intra-hepatic cholestasis, viral hepatitis

## Abstract

Alterations of methionine cycle in steatohepatitis, cirrhosis, and hepatocellular carcinoma induce MAT1A decrease and MAT2A increase expressions with the consequent decrease of S-adenosyl-L-methionine (SAM). This causes non-alcoholic fatty liver disease (NAFLD). SAM administration antagonizes pathological conditions, including galactosamine, acetaminophen, and ethanol intoxications, characterized by decreased intracellular SAM. Positive therapeutic effects of SAM/vitamin E or SAM/ursodeoxycholic acid in animal models with NAFLD and intrahepatic cholestasis were not confirmed in humans. In in vitro experiments, SAM and betaine potentiate PegIFN-alpha-2a/2b plus ribavirin antiviral effects. SAM plus betaine improves early viral kinetics and increases interferon-stimulated gene expression in patients with viral hepatitis non-responders to pegIFNα/ribavirin. SAM prevents hepatic cirrhosis, induced by CCl4, inhibits experimental tumors growth and is proapoptotic for hepatocellular carcinoma and MCF-7 breast cancer cells. SAM plus Decitabine arrest cancer growth and potentiate doxorubicin effects on breast, head, and neck cancers. Furthermore, SAM enhances the antitumor effect of gemcitabine against pancreatic cancer cells, inhibits growth of human prostate cancer PC-3, colorectal cancer, and osteosarcoma LM-7 and MG-63 cell lines; increases genomic stability of SW480 cells. SAM reduces colorectal cancer progression and inhibits the proliferation of preneoplastic rat liver cells in vivo. The discrepancy between positive results of SAM treatment of experimental tumors and modest effects against human disease may depend on more advanced human disease stage at moment of diagnosis.

## 1. Introduction

**Figure 1:**
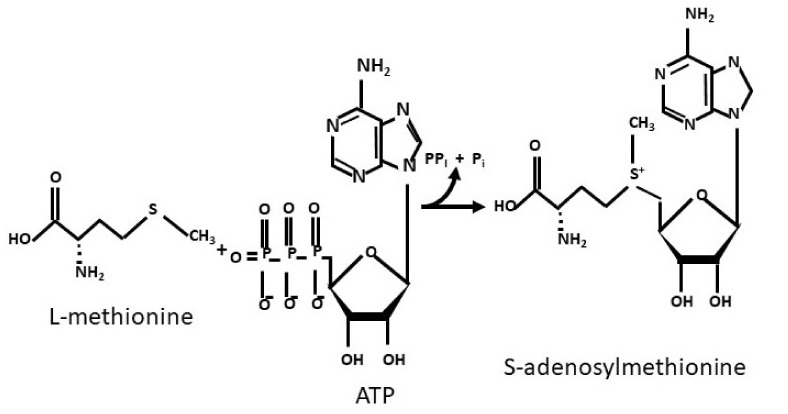
Synthesis of S-adenosylmethionine.

In 1951, Cantoni discovered the enzymatic formation of SAM, by the nucleophilic transfer of the adenosyl moiety of adenosinetriphosphate (ATP) to the sulphur atom of L-methionine [1] (Figure 1), which has been thereafter studied extensively in liver cancer [2,3].

**Figure 2:**
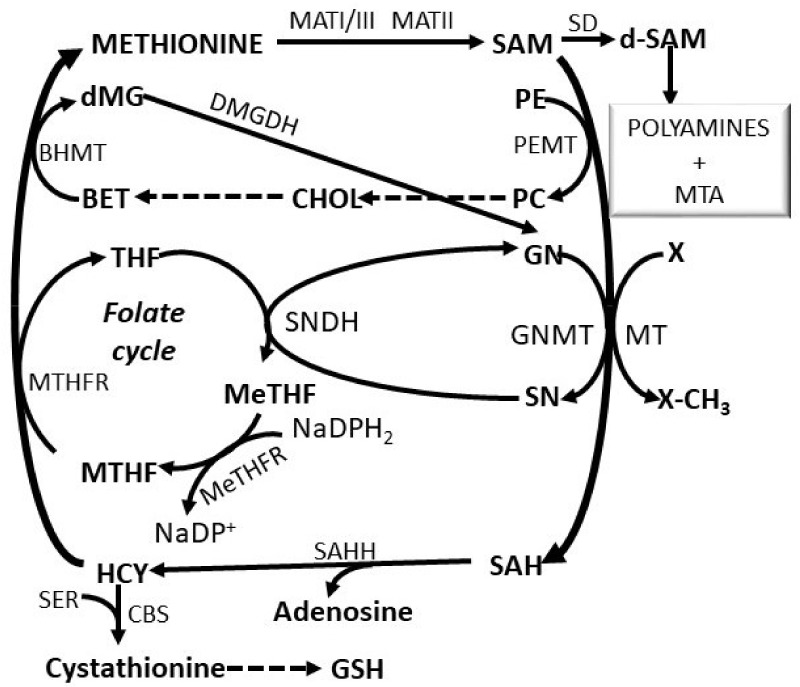
Metabolic cycles involved in methionine metabolism. *Substrates*: BET, betaine; CHOL, choline; DMG, dimethylglycine; dSAM, decarboxylated S-adenosylmethionine; GN, glycine; GSH, reduced glutathione; HCY, homocysteine; MeTHF, 5,10-methylenetetrahydrofolate; MTA, 5-methylthioadenosine; MTHF, 5-methyltetrahydrofolate; SAH, S-adenosylhomocysteine; SAM, S-adenosylmethionine; SN, sarcosine; THF, tetrahydrofolate. *Enzymes*: BHMT, betaine homocysteine methyltransferase; DMGDH, dimethylglycine dehydrogenase; GNMT, glycine n-methyltransferase; MATI/III, methyladenosyltransferase I/III; MATII, methyladenosyltransferase II; MeTHFR 5,10-methyltetrahydrofolate reductase; MT, various methyltransferases; PEMT, phosphatidylethanolamine N–methyltransferase; SAHH, S-adenosylhomocysteine hydroxylase; SD, SAM decarboxylase.

SAM is the first product of the “methionine cycle” and is implicated in the synthesis of polyamines and in the transsulfuration pathway leading to homocysteine and reduced glutathione (GSH) biosynthesis (Figure 2). SAM is synthesized from methionine and ATP in a reaction catalyzed by methionine adenosyltransferases (MATs) [4]. These enzymes are encoded by two genes, *MAT1A* and *MAT2A. MAT1A* encodes the α1 subunit of the tetramer MAT(α1)_4_ (MATI) and of the dimer MAT(α1)_2_ (MATIII). *MAT2A* encodes the α2-subunit of the MATII isoform, widely distributed. *MAT1A* is expressed in adult liver while *MAT2A* is prevalently expressed in fetal liver [5]. MATI and MATIII isozymes have intermediate (23 μM–1 mM) and high (215 μM–7 mM) Km for methionine, respectively. Thus, the physiological liver SAM level (60 μM) has low inhibitory effect on MATI and stimulates MATIII activity [4,5]. MATII has the lowest Km for methionine (4–10 pM) and is inhibited by the reaction product [5]. A third gene, *MAT2B*, encoding the subunit (β), regulates MATII by lowering its Km for methionine and Ki for SAM [6]. Therefore, the association of the β-subunit with the α2-subunit renders MATII more liable to be inhibited by SAM [6].

SAM may be decarboxylated by a specific decarboxylase. Decarboxylated SAM (dSAM) (Figure 2) is used for the synthesis of polyamines and 5′-methylthioadenosine (MTA). The latter, after transformation to methylthioribose by a specific nucleosidase, may be further used in the “salvage pathway” of methionine re-synthesis [7]. Furthermore, SAM plays an essential role as methyl-donor for the methylation reactions during which it is transformed to S-adenosylhomocysteine (SAH). SAH, a strong inhibitor of transmethylations, is transformed to homocysteine (HCY) by a specific hydroxylase (Figure 2). HCY may be transformed by a synthetase to cystathionine, a precursor of GSH, or is methylated for the resynthesis of methionine (Figure 2). This resynthesis, catalyzed by betaine homocysteine methyltransferase, may be coupled to the Bremer pathway [8,9] for the synthesis of phosphatidylcholine from phosphatidylethanolamine by phosphatidylethanolamine methyltransferase (PEMT). Alternatively, methionine resynthesis may be coupled to the folate cycle that provides methyl groups [10,11] during the synthesis of 5-tetrahydrofolate (THF) from 5-methyltetrahydrofolate (MTHF), catalyzed by a synthetase. THF is transformed to 10-methylenetetrahydrofolate (MeTHF), a precursor of MTHF, by methyltetrahydrofolate reductase, in a reaction coupled with the resynthesis of glycine from sarcosine (Figure 2). The methionine cycle plays a fundamental role in the cellular metabolism and the alteration of its functionality causes important disorders linked to modification of DNA methylation and gene expression, redox imbalance and metabolic reprogramming in liver and brain [12,13,14,15].

## 2. The Methionine Adenosyltransferase Switch

Liver cirrhosis and HCC of rodents and humans are characterized by a decrease of MAT1A expression and a rise in MAT2A expression with a consequent decrease of MAT1A:MAT2A ratio (the so-called MAT1A/MAT2A switch) [16]. MATI/III downregulation, consequent to the oxidation of cysteine residue in the ATP binding site, and GSH fall occur in cirrhotic liver [17,18]. SAM administration reconstitutes the GSH pool, protects MATI/III [17,18], and inhibits liver fibrosis in rats and humans [2,17,18,19,20,21]. Due to its inhibition by the reaction product, MATII upregulation does not compensate for MATI/III fall. Consequently, the SAM decrease in rapidly growing cancer cells depends on the diminution of MATI/III:MATII ratio, the increase in SAM decarboxylation for polyamine synthesis [22] and the inhibition of BHMT activity by SAM [12,14,23]. On the whole, these findings indicate that the MAT1A/MAT2A switch and the decrease in SAM level are implicated in hepatocarcinogenesis. Therefore, chronically SAM deficient MAT1A-KO mice, even in the presence of MAT2A induction, undergo hepatomegaly without histologic abnormalities at three months of age, steatosis of 25–50% of hepatocytes and infiltration of mononuclear cell in periportal areas, at eight months, and HCC at 18 months of age [24].

## 3. Regulatory Mechanisms of the Methionine Cycle

**Figure 3:**
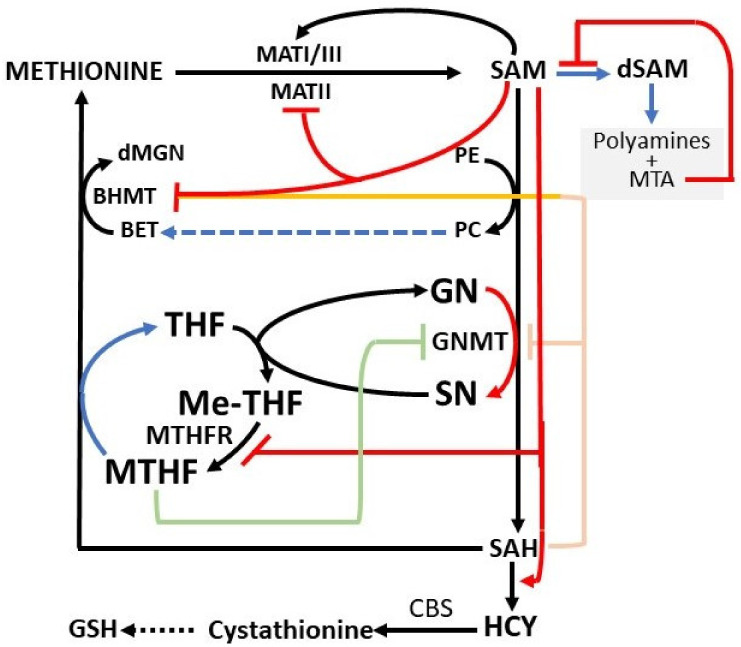
SAM and SAH long-range interactions. BET: betaine; BHMT: betaine homocysteine methyltransferase; dMGN: dimethylglycine; HCY: homocysteine; MATI/III: methionine adenosyltransferase I/III; MATII: methionine adenosyltransferase II; GN: glycine; GNMT: glycine methyltransferase; GSH, reduced glutathione; Me-THF: 5,10-methylenetetrahydrofolate; MHMT: methyltetrahydrofolate homocysteine methyltransferase; PC: phosphatidylcholine; PE: phosphatidylethanolamine; SAH: S-adenosylhomocysteine; SAM: S-adenosylmethionine; SN: sarcosine. Arrows indicate activation; blunt arrows indicate inhibition.

The methionine and folate cycles exert various regulatory functions (Figure 3). SAM “long range interactions” inhibit MATII and activate MATI/III, thus impeding the MATI/III/MATII switch [18].

Furthermore, SAM inhibits BHMT [11,12,13] and MTHFR [14,15] and then the methionine re-synthesis and the purine and deoxythymidylate synthesis, with a consequent rise of homocysteine and GSH synthesis. The inhibition of MeTHFR by SAM causes the decrease of free MTHF and is followed by the dissociation of GNMT-MTHF complex [13,17]. In addition, GNMT regulates the SAM/SAH ratio and SAM-dependent methyl transfer reactions. The Km value of GNMT for SAM is relatively high, and GNMT is poorly inhibited by SAH because its Ki value for SAH (35–80 μM) is higher than that for other SAM-dependent methyltransferases that are inhibited by SAH [8,13]. Thus, GNMT is active at SAM and SAH physiological levels (0.1–0.2 μmol/g and 0.02–0.06 μmol/g of the liver, respectively). Its activity may influence SAM/SAH ratio and the activity of other methyltransferases. Besides, GNMT protein binds folate and is inhibited by MTHF [8,9,10,11,16]. The rise of free GNMT avoids excessive SAM increase. On the contrary, due to the decrease in SAM concentration, MeTHFR inhibition is released, MTHF availability rises, and the free GNMT falls. Therefore, by increasing the cellular folate level and thus the MTHFR-dependent SAH remethylation, GNMT acts as a “salvage pathway” [16,25,26,27,28,29,30].

## 4. The Deregulation of Methionine Metabolism in Preneoplastic and Neoplastic Liver

The decrease of *MAT1A* expression in alcoholic hepatitis, liver cirrhosis and HCC [16,31] on the whole depends, transcriptionally, on the methylation of CpG of the *MAT1A* promoter and the deacetylation of histone H4 and, post-transcriptionally, on the interaction of MAT1A mRNA with the AUF1 protein that increases its decay [32]. In contrast, the upregulation of *MAT2A* gene in HCC depends on the promoter hypomethylation and histone H4 acetylation, and the increased stability of MAT2A mRNA due to its interaction with HuR (human antigen R) protein [32,33,34]. Furthermore, different activating factors, including Sp1, c-Myb (avian myeloblastosis viral oncogene homolog), nuclear factor kappa B (NF-kB), and AP-1 concur to MAT2A transcriptional upregulation in HCC [35,36].

MATα2 regulates the expression of *BCL*-*2* in the RKO human colon cancer cell line and in the HepG2 liver cancer cell line [37]. In both cell lines MATα2 activates *BCL*-*2* gene transcription by binding to its promoter. It also directly interacts with BCL-2 protein enhancing its stability. These MATα2 effects involve the ubiquitin-conjugating enzyme 9 required for the sumoylation of MATα2 at K340, K372, and K394, necessary for MATα2 stability [38]. *MAT2B* encodes the MAT2β regulatory subunit that modulates the activity of—encoded isoenzyme *MAT2A.* The mechanisms regulating of MAT2β expression are poorly known. MAT2β promoter is activated by Sp1 [38]. In HCC, two dominant splicing variants of *MAT2B*, V1, and V2 are upregulated. TNFα (tumor necrosis factor α) and leptin activate the *MAT2B* V1 promoter while SAM inhibits it by mechanisms involving ERK and AKT signaling [39]. MATβ2 protein regulates various other proteins [39,40,41], including GIT1 that is activated by MATβ2 [42]. GIT1 activates the RAS/RAF/MEK1/2/ERK1/2 signaling, thus inducing liver and colon cancer cells proliferation [43].

HuR is a key regulator of cellular mRNAs containing adenylate/uridylate–rich elements (AREs). SAM regulates HGF (hepatocyte growth factor)-mediated hepatocyte proliferation through a mechanism implicating the activation of LKB1/AMPK/eNOS cascade [44]. It also regulates the cytoplasmic HuR function in cancer cells via AMP-activated kinase [45]. mRNA-binding proteins are involved in the post-transcriptional deregulation of gene expression. Thus, AUF1 increases mRNA decay while HuR selectively binds to AU-rich elements increasing mRNA stability [32,46,47,48,49]. Interestingly, Mat1A decrease, Mat1A:Mat2A switch, and low SAM levels are associated with CpG hypermethylation and histone H4 deacetylation of Mat1A promoter, and prevalent CpG hypomethylation and histone H4 acetylation of Mat2A promoter. In the HCC of genetically resistant BN rats, very low changes in the Mat1A:Mat2A ratio, CpG methylation, and histone H4 acetylation occur. The levels of AUF1 protein, which destabilizes MAT1A mRNA, Mat1A-AUF1 ribonucleoprotein, HuR protein, which stabilizes MAT2A mRNA, and the Mat2A-HuR ribonucleoprotein increase in HCC of genetically susceptible F344 rats and in human HCC with poorer prognosis (HCCP), and undergo low/no increase in BN HCC and human HCC with better prognosis (HCCB) [50]. In human HCC, Mat1A:MAT2A expression and MATI/III:MATII activity ratios are negatively correlated with cell proliferation and genomic instability, and positively correlated with apoptosis and DNA methylation. Forced MAT1A overexpression in the liver carcinoma cell lines HepG2 and HuH7 results in a rise in SAM level, inhibits cell proliferation and induces apoptosis [50]. These changes are associated with the down-regulation of *Cyclin D1*, *E2F1*, *IKK*, *NF-kB*, and the antiapoptotic *BCL2* and *XIAP* genes, and with the up-regulation of *BAX* and *BAK* proapoptotic genes. Moreover, SAM treatment of rats, during the development of preneoplastic foci, impedes *NF-kB* activation [51] and stimulates the expression of the oncosuppressor gene *PP2A* (protein phosphatase 2) and other oncosuppressors that inhibit the progression of preneoplastic nodules to HCC. Accordingly, *PP2A* is underexpressed in human and rat HCCs with low SAM content, high pAKT and pERK expression and proliferation rate [18,51,52,53].

Interestingly, in a SAM-deficient cell line, isolated from an from an HCC of MAT1A-KO mouse LKB1 expression is required for cell survival mouse, LKB1 expression is required for cell survival [54]. LKB1 regulates the AMPK and mTORC2 and controls the apoptotic response through the phosphorylation and retention of p53 in the cytoplasm and the regulation of HAUSP and HuR nucleocytoplasmic shuttling [54].

Recent observations indicate the existence of relationships between some miRNAs and the expression of MATs. The individual knockdown of miR-664, miR-485-3p, and miR-495 in Hep3B and HepG2 cells induces MAT1A expression. Hep3B cells tumorigenesis in nude mice is decreased by the stable overexpression of these miRNAs and increased by their knockdown [55,56], suggesting that their upregulation contributes to hepatocarcinogenesis by lowering MAT1A expression. These observations indicate that both transcriptional and post-transcriptional mechanisms contribute to MAT1A/MAT2A switch and SAM decrease during hepatocarcinogenesis, and suggest that MAT1A/MAT2A switch and SAM reduction may have a prognostic value for hepatocarcinogenesis. Recent research indeed showed that miR-21-3p lessens *MAT2A* and *MAT2B* expression in HepG2 cells by targeting their 3′-primer untranslated regions (3′-UTRs) and inhibits cell growth [56]. Furthermore, miR-203 expression is inversely correlated with MAT2A and MAT2B expression and the expression of markers of HCC proliferation and aggressiveness [57]. MiR-203 expression is genetically regulated and contributes to patients’ outcomes [57] and MiR-203 transfection in liver cancer cells targets the 3′-UTR of *MAT2A* and *MAT2B* genes and strongly inhibits their expression. These findings suggest that miR-203 expression could predict HCC prognosis and may function as a biomarker for patient stratification and drug selection.

## 5. SAM Inhibitory Effects

Different pathologic conditions leading to a decrease of the SAM cellular content are antagonized by the administration of exogenous SAM. Thus, SAM antagonizes rat liver damage induced by galactosamine [58] or acetaminophen [59] prevents the steatosis induced by ethanol in rats and mice [18,19,20,60,61]. These effects largely depend on the capacity of SAM to preserve an adequate GSH content and the transport of GSH into mitochondria [62,63]. Indeed, the treatment with SAM of rats, subjected to the intraperitoneal injection of CCl_4_, causes a decrease of the incidence of hepatic cirrhosis and of the liver SAM/SAH ratio and induces an increase of serum homocysteine thus preventing the decrease of liver folates and GSH [63,64]. SAM inhibits collagen synthesis by human fibroblasts in vitro [65], protects against the alcohol induced acute hepatotoxicity in rats, mice, and baboons and decreases the synthesis of collagen by cultured fibroblasts [65,66,67,68,69,70]. In ethanol intoxicated rats, the decreased synthesis of phosphatidylcholine by the phosphatidylethanolamine methyltransferase (Bremer pathway) [71] is contrasted by SAM [8,9]. Furthermore, the treatment with SAM of the hepatocytes isolated from fatty liver induced by a choline-deficient diet may activate the synthesis of phosphatidylcholine by the CDP-choline pathway (Kennedy pathway) [72]. It was indeed demonstrated that a high cellular SAM pool allows the substitution of the transmethylation by the Kennedy pathway to supply the phosphatidylcholine moiety of lipoproteins [73].

The induction of liver cancer in rats fed adequate diet causes a fall in liver SAM content and SAM/SAH ratio that persists in dysplastic nodules (DN) and HCC even after the interruption of carcinogen administration [2,3,23,74]. A decrease of SAM/SAH ratio was also found in human HCC [75]. The administration of SAM to carcinogen-treated rats prevents hepatocarcinogenesis [76,77,78,79,80] and SAM intravenous infusion inhibits the orthotropic HCC development in liver of rats injected with H4IIE human HCC cells [80]. However, the infusion of SAM for 24 days to rats after the development of tumors is without effect, probably because SAM accumulation is prevented by the compensatory induction of GNMT [81]. The inhibition by SAM and MTA of colon carcinogenesis has also been observed and tentatively attributed to their therapeutic effect on the chronic inflammation that represents a major risk factor for this cancer [81]. In in vitro growing human colon cancer cells MAT2A is overexpressed, its inhibition by SAM and MTA blocks the growth [82]. SAM treatment of rats during the development of preneoplastic and neoplastic liver lesions inhibits the proliferation and induces apoptosis of preneoplastic cells [75,76,83,84]. Furthermore, an increase in SAM content, associated with the inhibition of proliferation, occurs in Huh7 liver tumor cells transfected with *MAT1A* [83].

Ornithine decarboxylase (ODC) is overexpressed in DNs and HCCs of carcinogen-treated F344 rats [21,84]. Early studies on HCC chemoprevention by SAM showed a decrease of ODC activity and polyamine synthesis in preneoplastic and neoplastic liver lesions [21,78]. MTA, an end-product of polyamine synthesis, inhibits SAM decarboxylase, thus limiting this synthesis. Moreover, MTA prevents lipid peroxidation and fibrogenesis induced by carbon tetrachloride [85]. It must be considered, however, that SAM treatment leads to the accumulation of only moderate amounts of MTA, probably because of MTA implication in the “salvage pathway” leading to methionine synthesis [86]. Interestingly, SAM and MTA treatments of CCl_4_-intoxicated rats were found to maintain a high GSH level [87]. This suggests that an antioxidative effect concurs to the HCC chemopreventive action of these compounds [88,89]. It must be considered, however, that SAM exerts an anticarcinogenic effect higher and independent of that of MTA [76].

**Figure 4:**
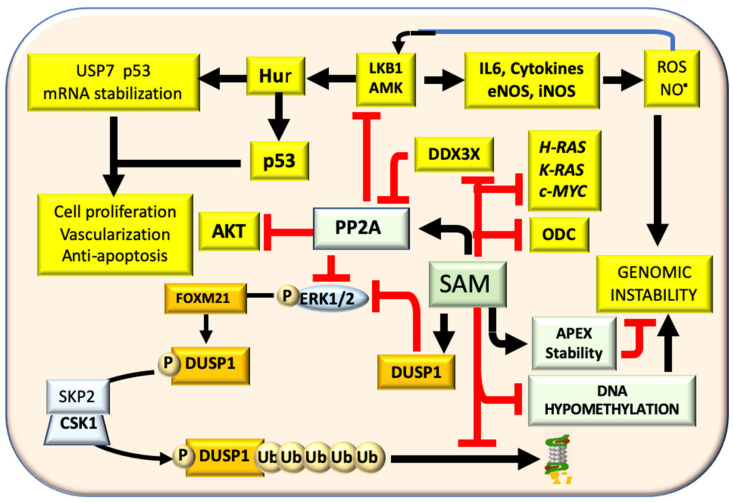
Effects of SAM on signal transduction pathways. SAM inhibits ODC activity and H-RAS, K-RAS, c-MYC expression. Through the inhibition of LKB1/AMPK axis, SAM controls p53 phosphorylation and cell growth and survival by inducing PP2A expression that dephosphorylating inactivates AKT. Moreover, PP2A activation and DUSP1 stabilization inhibit the RAS/ERK pathway. DUSP1 phosphorylation at the ser296, induced by the ERK1/2 target FOXM1, allows its ubiquitination by the SKP2/CKS1 ubiquitin ligase. This is followed by the proteasomal degradation of DUSP1. SAM enhances the DUSP1 inhibitory action by increasing the transcription of DUSP1 mRNA and by inhibiting the DUSP1 proteasomal degradation. The inhibition of LKB1/AMPK decreases eNOS and iNOS activity and ROS and NO, and thus the genomic instability that is also reduced by the decrease of DNA hypomethylation.

Early studies on the influence of SAM on signal transduction pathways showed that SAM injection to carcinogen-treated rats, during the development of preneoplastic liver lesions, inhibits ODC activity [84] and c-*myc*, *H-ras*, and *K-ras* expression [83] (Figure 4).

This inhibition could depend on the MTA production during polyamine synthesis [2] and the protooncogenes inhibition could be attributed to the reversion of their hypomethylation. SAM also causes a decrease of ERK1/2 activity by inducing the ERK1/2 inhibitor DUSP1 [90,91] (Figure 4). In fast growing DNs and HCCs of F344 rats, genetically susceptible to hepatocarcinogenesis, and in human HCCs with poor prognosis (HCCP) ERK1/2 overexpression is associated with a low expression of DUSP1 [91]. ERK1/2 phosphorylates DUSP1 allowing its ubiquitination by the SKP2-CKS1 ubiquitin ligase followed by its proteasomal degradation [91] (Figure 4). ERK1/2 sustains the activity of SKP2-CKS1 via its target FOXM1, which mediates the ERK1/2 effects on cell cycle, cell survival, and angiogenesis [92]. In accordance with these findings, the livers of MAT1A-KO mice and cultured mouse and human hepatocytes have low levels of ERK1/2 mRNA and protein [90]. SAM inhibits the proteasomal degradation of DUSP1 and its administration to MAT1A-KO mice increases the expression and causes a decrease in the ERK1/2 activity [90]. The ERK activity was unrestricted during HCC progression by activating the ubiquitin-mediated proteolysis of its specific inhibitor DUSP1 [91]. Thus, DUSP1 may represent a valuable prognostic marker and ERK, CKS1, or SKP2 potential therapeutic targets for human HCC [92]. FOXM1 upregulation is associated with the acquisition of a susceptible phenotype in rats and influences human HCC development and prognosis [93]. FOXM1 expression is also sustained by the TNF-/HIF-1 axis [93,94]. The hypoxia may redu ce the SAM level of HCC cells by binding HIF-1 to the MAT2A promoter [95]. The activation of PP2A by SAM has other important consequences. PP2A inhibits LKB1/AMPK, AKT, and ERK [96,97]. Lkb1 induces the HuR nuclear/cytoplasmic translocation that stabilizes Cyclins mRNA thus inducing cell proliferation [98]. Furthermore, LKB1 induces the hyperphosphorylation and cytoplasmic retention of p53 allowing its interaction with the de-ubiquitinating enzyme, USP7, that blocks p53 inhibition by MDM2 [99]. Furthermore, cytosolic HuR stabilizes p53 and USP7 mRNAs [100]. SAM blocks LKB1/AMPK activation [100] (Figure 4). Notably, cytoplasmic staining of p53 and p-LKB1 (Ser428) occurs in NASH and HCC of MAT1A-KO mice and in liver biopsies of human HCC induced by ASH and NASH [54]. However, these observations contrast with the LKB1 loss found in different tumors, including HCC [44]. LKB1 is considered an oncosuppressor gene [101], and AMPK activated by LKB1 inhibits AKT signaling [102]. The downregulation of the AMPK is present in undifferentiated HCC [103].

The DNA damage induced by the reactive nitrogen species, produced via iNOS/eNOS during chronic hepatitis, may be involved in carcinogenesis [104]. SAM hepatoprotective effect may be also mediated by the modulation of NO production [29,105,106,107,108,109,110]. The variations of SAM levels may modify the mitogenic stimulus of HGF through the modulation of intracellular SAM levels. The mitogenic response to HGF is repressed by the inhibition of NO synthase-2 a process overwhelmed by the addition of an NO donor. This effect depends on the intracellular SAM levels. Accordingly, SAM inhibits HGF-induced cyclin D1 and D2 expression, activator protein 1 induction, and hepatocyte proliferation. Thus, NO may switch hepatocytes into a growth factor-responsive state through the down-regulation of SAM levels. This effect depends on the intracellular SAM levels [110]. The rise of NO production in hepatocytes after PH or treatment with growth factors inhibits MATI/III by S-nitrosylation of cysteine 121 and, consequently, reduces SAM cellular levels. This preserves HGF-induced hepatocyte proliferation from the inhibitory action of SAM [110].

Furthermore, convincing evidence indicates that MTA may influence hepatocarcinogenesis independently of its conversion to SAM. Oral MTA administration to Mdr2(-/-) mice for three weeks reduces liver inflammation and fibrosis [111]. It was found that MTA may have multiple molecular and cellular targets including the inhibition of inflammatory and profibrogenic cytokines, and the attenuation of cultured myofibroblast activation and proliferation. Downregulation of JunD and cyclin D1 expression in myofibroblasts may be important regarding the mechanism of action of MTA. This compound could be a good candidate to be tested for the treatment of (biliary) liver fibrosis.

The mitogenic response to HGF (hepatocyte growth factor) is reduced when inducible NO synthase is inhibited, a process overwhelmed by the addition of a NO. donor. This effect depends on the methionine concentration in the culture medium as well as on the cellular SAM level. NO modulates inducible nitric oxide synthase gene expression [112]. Further, MTA administration to murine macrophage RAW 264.7 cells, and isolated rat hepatocytes treated with pro-inflammatory cytokines completely prevented LPS-induced lethality. This was associated to the suppression of circulating (TNF-α), inducible NO synthase (iNOS) expression, and the stimulation of IL-10 synthesis. These responses to MTA were also found in LPS-treated RAW 264.7 cells. MTA prevented the transcriptional activation of iNOS by pro-inflammatory cytokines in isolated hepatocytes, and the induction of cyclooxygenase 2 (COX2) in RAW 264.7 cells. In these cells MTA inhibited the activation of MASPK (p38 mitogen-activated protein kinase), c-jun phosphorylation, degradation of IkB-α (inhibitor kappa B-α) and NFKB (nuclear factor kappaB) activation. These effects were independent of the metabolic conversion of MTA into SAM [112].

The interaction of MTA with the cAMP signaling pathway, is involved in its anti-inflammatory effect. The methylation of RAF proteins by PRMT5 (protein arginine methyltransferase 5) leads to a decrease of ERK1/2 phosphorylation [113]. The PRMT5-dependent methylation enhances the degradation of active CRAF and BRAF and deceases their activity. The inhibition of PRMT5 or the expression of RAF mutants (that are not methylated) affects the amplitude and duration of ERK phosphorylation in response to growth factors and redirects from the proliferation to differentiation the response of PC12 cells to EGF.

### SAM Metabolism and Epigenetic Regulation

Epigenetic events, involved in changes in gene expression, are important for tumor progression, and variation in genes involved in epigenetic mechanisms could be important in cancer susceptibility. A large case-control study identified 63 single nucleotide polymorphisms (SNPs) that were genotyped in 75 human cancer cell lines from different tumor types to assess the existence of an association between them and six epigenetic measures. No statistically significant association was found. However, a trend was observed: homozygotes for the rare alleles of the EHMT1, EHMT2, and PRDM2 had a mean value for both trimethylation of K9 and K27 of histone H3 remarkably different to the homozygotes for the common alleles [114]. These preliminary observations suggest the possible existence of a functional consequence of harboring these genetic variants in histone methyltransferases. Tagging single-nucleotide polymorphisms in antioxidant defense enzymes and susceptibility to breast cancer.

The hypermethylation status of the *p16INK4a* (*p16*) gene promoter was analyzed in normal-appearing mucosa of patients with colorectal cancer. The hypermethylation of p16 was associated with reduced survival. Germ line polymorphisms in methylenetetrahydrofolate reductase (MTHFR), methionine synthase (MTR) and methionine synthase reductase (MTRR) were analyzed. No differences in cancer-specific or disease-free survival of stage I-III patients according to polymorphic variants and in cancer-specific or disease-free survival were detected in patients sub-grouped according to the MTHFR or MTR genotype and dichotomized by *p16* hypermethylation status in mucosa. Patients with the MTRR 66 AA/AG genotypes had a worse survival when the mucosa was positive for p16 hypermethylation. In contrast, there was no difference in survival among patients with the MTRR 66 GG genotype stratified by p16 hypermethylation status. These results indicate a relationship between genetic germ-line variants of the MTRR gene and p16 hypermethylation in mucosa, which may affect the clinical outcome of patients with colorectal cancer [115].

## 6. SAM and Human Disease

### 6.1. Alcoholic Liver Disease

The alcoholic liver disease (ALD) is the prevalent chronic liver disease worldwide that may progress from alcoholic fatty liver (AFL) to alcoholic steatohepatitis (ASH). Chronic ASH may lead to fibrosis and cirrhosis and, in some patients, to HCC. Furthermore, severe ASH can lead, in a subset of individuals, to alcoholic hepatitis with liver failure and high mortality. The variability of ALD phenotype probably depends on genetic, epigenetic and non-genetic factors. The pathogenesis of ALD includes hepatic steatosis, oxidative stress, acetaldehyde toxicity, and inflammation induced by cytokines and chemokines. As a consequence of ETOH toxicity, excessive alcohol consumption induces progressive liver injury including steatosis, steatohepatitis and cirrhosis. ROS production is implicated in these effects [70,116]. In the patients with ALD, the deficiencies of nutrients essential for normal methionine metabolism have deleterious effects since they impair the remethylation of homocysteine, by MTHFR and BHMT, and GSH synthesis, thereby decreasing the defenses against oxidative stress.

A two-year Spanish multi-center study examined the effect of 1.2 g/day of oral SAM in 123 patients with cirrhosis caused by ALD [117]. This study found that mortality decreased from 30% in the placebo group to 16% in SAM-treated but this decrease was not statistically significant unless the patients with more advanced disease were excluded. Long-term treatment with SAM improved the survival or delayed liver transplantation of the patients with alcoholic liver cirrhosis, especially of those with less advanced liver disease [117].

SAM plays a role in numerous cellular reactions. Because of the decrease in its synthesis in various liver diseases, different studies have considered the effects of the reconstitution of SAM cellular pool by SAM therapy. Some randomized clinical trials have considered this therapy in the treatment of specific human diseases. SAM increases intra-hepatic GSH and improves clinical biochemistry in patients with alcoholic and non-alcoholic liver disease, it was demonstrated that SAM increases intra-hepatic GSH and improves clinical biochemistry [118,119,120,121]. It must be considered that the ethanol metabolite acetaldehyde displaces the active form of pyridoxal phosphatase (vitamin B6) from its hepatic binding site [122,123]. The deficiency of vitamin B6, prejudices homocysteine remethylation by MTHFR and BHMT (Figure 2) and its metabolism to form GSH, thus impairing the defenses against oxidative stress. The consequent increase in hepatocellular homocysteine will alter the catalytic equilibrium of the reversible enzyme SAH hydrolase, and consequently the SAM:SAH ratio thus inhibiting many SAM dependent methylation reactions.

In a study [124] on a cohort of 37 patients with alcoholic liver disease treated with 1.2 g of SAM by mouth for 24 weeks, the entire cohort showed an overall improvement of AST, ALT, and bilirubin levels at the end of the treatment. However, there were no differences between the SAM-treated and controls in any clinical or biochemical parameters and in liver histopathology scores for steatosis, inflammation, fibrosis, and Mallory-Denk hyaline bodies. It was concluded that SAM was no more effective than placebo in the treatment of alcoholic liver disease. It must be considered, however, that in this relatively small study only a six-month follow-up was performed.

In a study according to the intention-to-treat methodology, data provided by the Cochrane Collaboration, nine randomized clinical trials including a heterogeneous sample of 434 patients with ALD, were identified and analyzed [125]. Eight out of nine trials were placebo controlled. Only one trial including 123 patients with alcoholic cirrhosis used adequate methodology, no significant effects of SAM were found on all-cause mortality, liver-related mortality or liver transplantation or complications. SAM was not associated with non-serious adverse events. It was concluded that there was no evidence supporting or refuting the use of SAM for patients with ALD. In conclusion, no evidence in support or denial of SAM therapy is available. More long-term, high-quality randomized trials studying patients with ALD, treated with oral/parenteral administration of SAM versus placebo, are necessary for SAM may be recommended for clinical practice.

### 6.2. Non-Alcoholic Fatty Liver Disease

NAFLD is a metabolic syndrome affecting people that do not abuse alcoholic beverages. It is associated with obesity, insulin resistance or type 2 diabetes mellitus, and dyslipidemia, and is characterized by the development of steatosis, non-alcoholic steatohepatitis (NASH), and cirrhosis [126,127,128]. Obesity, diabetes, insulin resistance, sedentary lifestyle, and Western diet generally underlay NAFLD, a common liver disease in developed countries. NAFLD may further progress to NASH, fibrosis, cirrhosis, and HCC. The hepatic steatosis and extrahepatic clinical manifestations, including adipose tissue inflammation and gastrointestinal imbalances determine the evolution of NAFLD to NASH. Recent evidence indicates that gut-derived bacterial toxins, the activation of the innate immune system, and oxidative stress are common pathogenic mechanisms determining the progression of alcoholic liver disease and NASH [129,130]. Recent research has found that cholesterol metabolism is closely related to the pathogenesis and severity of NASH [131]. A “multi-parallel hit” hypothesis has been proposed. Cholesterol affects membrane fluidity and membrane protein function through genetic factors and can also induce unfolded protein response and generate toxic oxysterol. Free cholesterol can activate hepatic Kupffer and stellate cells to produce inflammatory cytokines and collagen. The formation of cholesterol crystallization and crown-like structures can damage liver cells and activate Kupffer cells. Hepatocytes alteration by free cholesterol accumulation is also induced by the by disruption of mitochondrial and endoplasmic reticulum membrane integrity, causing mitochondrial oxidative injury, promoting toxic oxysterols generation, and inducing adipose tissue dysfunction. Accumulation of oxidized LDLs may activate Kupffer and hepatic stellate cells with consequent inflammation and fibrogenesis. Furthermore, the damage induced by cholesterol also depends on elevated cholesterol uptake from circulating lipoproteins and reduced cholesterol excretion. Extensive dysregulation of cellular cholesterol homeostasis by nuclear transcription factors sterol regulatory binding protein (SREBP)-2, liver X-receptor, (LXR)-α and farnesoid X receptor (FXR) plays a key role in hepatic cholesterol accumulation in NASH [132].

Different experimental models of NASH, generally based on feeding methionine and choline-deficient diet, have been developed in mice [133,134,135,136,137]. Apoptosis, associated with p53 activation and TRAIL receptor expression, occurs in experimental NASH [134]. Different studies on NAFLD pathogenesis have shown that the initiating events of NAFLD are the development of obesity with insulin resistance and type 2 diabetes, increased hepatic free fatty acid flux, increased oxidative stress during fatty acid oxidation, activation of the innate immune system with cytokine release followed by hepatic fibrosis [126,132,136].

SAM could influence NAFLD pathogenesis because of its role in the synthesis of GSH and of phosphatidylcholine, by the Bremer pathway that is involved in VLDL assembly and hepatic triglyceride export. Apoptosis associated with p53 activation and TRAIL receptor expression occurs in experimental NASH [126]. Oxidative stress (CYP2E1 induction), lipid peroxidation, cytokines and principally TNF-alpha are involved in the progression of steatosis to steatohepatitis [132,135].

Prolonged consumption by rodents of a methionine and choline deficient diet, leads to a sharp decrease of liver SAM and VLDL and induces fibrosing steatohepatitis [136]. Similar alterations have been described in MATO-KO mice, a NASH-HCC animal model that in the absence of Mat1a cannot synthesize SAM and presents steatosis involving 25–50% of hepatocytes and mononuclear cell infiltration in periportal areas, at eight months, and HCC at 18 months of age [23,138,139,140]. In a human study [130] in which the rates of remethylation of homocysteine and transmethylation of methionine were evaluated in 15 patients with NASH, compared to 19 healthy controls, the inactivation of MATI/III and increased oxidative stress reduced significantly the synthesis of methionine by homocysteine remethylation [141].

NAFLD is the prevalent liver disease worldwide, and there is no approved pharmacotherapy. The first approach to the therapy of NAFLD targeted the hepatic fat accumulation, by modulating the peroxisome proliferator-activator receptors, the farnesoid X receptor axis and the de novo lipogenesis. A second therapeutic target was the control of oxidative stress, inflammation and apoptosis. A third target was the intestinal microbiomes and metabolic endotoxemia. The final target was hepatic fibrosis, which is strongly associated with all-causes of liver-related mortality in NASH. Some research reported promising results of the anti-oxidant vitamin E associated with pioglitazone in NASH therapy [142]. A study group on 119 children with NAFLD showed that in comparison with metformin, vitamin E is more influential in remission; however both are efficient in treatment of fatty liver [143]. Another study suggest that metformin treatment is more effective than dietary advice and vitamin E treatment in reducing insulin resistance, and also in ameliorating metabolic parameters such as fasting insulin and lipid levels, in obese adolescents having NAFLD [144].

A clinical benefit of SAM, as precursor of the antioxidant compound GSH, and of Betaine that provides methyl groups for the remethylation of homocysteine to methionine, precursor of SAM (Figure 2), was hypothesized. However, the positive results obtained in animal models and in a pilot study were not confirmed in a subsequent randomized placebo-control trial [145], which only demonstrated that betaine therapy increased the serum methionine and SAM contents but did not improve the histology that showed stabilization of steatosis compared to controls. Furthermore, it must be considered that the exit liver biopsy was not done in about 32% of the patients [146].

An interesting comparison between different treatments of NASH [147] showed that interventional clinical trials involving 18 different agents, alone and in combination, were identified. Pioglitazone was the only agent that showed consistent benefit and efficacy in clinical trials. Pentoxifylline, rosiglitazone, and ursodeoxycholic acid had both positive and negative results. There was also evidence for vitamin E that reduced steatosis, lobular inflammation, and aminotransferases and metformin.

### 6.3. SAM and Intra-Hepatic Cholestasis

Intra-hepatic cholestasis (IHC) develops as a consequence of a reduced sub-lobular bile flow caused by different conditions including hepatocellular damage induced by viral or alcoholic hepatitis, prolonged total parenteral nutrition, canalicular membrane alterations, produced by oral contraceptives, antibiotics, etc., genetic deficiencies of bile transporters, obstructions of canaliculi or ductules or/and ductopenia [148]. Some studies have shown that oxidative stress occurs in livers of humans with cholestasis [149]. In vitro studies demonstrated that bile acids kill hepatocytes [149], but this mechanism was of limited importance in a rat model in vivo. In this model the inflammatory response caused neutrophil accumulation and production of ROS, while the inhibition of ROS during cholestasis reduced the fibrosis [150]. Furthermore, in the experimental rat cholestasis [151], induced by bile duct ligation, there occurred an increase in plasma homocysteine, secondary to NO overproduction, and a decrease in liver SAM and SAH contents in precirrhotic stages and in secondary biliary cirrhosis. NO overproduction probably contributed to plasma increase of SAM and to liver SAM depletion after cholestasis [110].

Different multi-center, double-blind, placebo-controlled trials, showed that the oral SAM treatment (800–1600 mg/day) induced a significant decrease of the clinical biochemical indices of cholestasis and an improvement of symptoms of fatigue and pruritus [152,153,154,155]. In another study, a systematic review and meta-analysis of randomized controlled trials (RCTs) evaluated the effect and safety of ursodeoxycholic acid (UDCA), SAM and UDCA-SAM combination therapies for intrahepatic cholestasis of pregnancy (ICP). It was found that UDCA-SAM combination therapy is better than UDCA or SAM alone for improving the outcome of ICP without adverse effects [156].

In a comparison between SAM and Chinese Yinchenghao decoction (YCHD) for the treatment of ICP it was concluded that both treatments could be efficacious [157]. However, in a meta-analysis of all randomized controlled trials comparing UDCA, SAM, and their combination, using Pubmed, Embase, the Cochrane register of controlled trials, and the Science Citation Index of web of science, including 311 patients, it was found that UDCA decreased the pruritus score, the levels of total bile acids, and alanine aminotransferase levels more effectively than SAM and reduced the rate of preterm delivery for ICP. SAM and viral hepatitis [158].

### 6.4. SAM and Viral Hepatitis

Chronic infection with hepatitis C virus (HCV) affects 170 million people worldwide and is the leading cause of cirrhosis in North America. The recommended treatment consists in the administration of peginterferon-alpha-2b (PegIFN-alpha-2b) and/or peginterferon-alpha-2a plus ribavirin. Liver biopsies from patients with chronic hepatitis C virus (HCV) infection showed the impairment of the Interferon-alpha-induced DNA binding of STAT1 compared with controls [159]. This depended on the hypomethylation of STAT1 on arginine 31 that allowed its association with PIAS1, an inhibitor of STAT DNA binding. The overexpression of Protein Phosphatase 2A (PP2A) in liver extracts from HCV transgenic mice and in liver biopsies of patients with HCV leads to STAT1 hypomethylation, increase of its binding to PIAS1, and decrease of interferon-alpha-induced DNA binding of STAT1 [160]. However, many patients, especially if of African ancestry, are not cured by the treatment with PegIFN-alpha-2b or PegIFN-alpha-2a. In the attempt to identify the determinants of response to the treatment, it was found that the genetic polymorphism near the IL28B gene, encoding interferon-lambda-3 (IFN-lambda-3), is associated with about twofold change in response to treatment. The addition of SAM or of SAM and betaine [155,160,161] to pegIFNα/ribavirin improves the early viral kinetics and increases the interferon-stimulated gene expression in non-responders to previous therapy. Furthermore, in vitro experiments demonstrated that SAM and betaine inhibit interferon signaling and restore STAT1 methylation, thus improving the Interferon-alpha-induced DNA binding of STAT1 and enhancing the antiviral effect of IFNα in cell culture.

At present the mechanisms determining the response to the treatment with Pegylated IFNα/Ribavirin are not completely known. A possible mechanism could be the HCV-induced viral interference with IFNα and JAK-STAT signaling, determined by the hypomethylation of STAT1 that facilitates the contact of STAT1 with its inhibitor PIAS1 (protein inhibitor of activated STAT1) [153]. In addition, two SNPs near the gene IL28B on chromosome 19 were found to be strongly associated with non-viral response [154,155].

In a study on the molecular mechanism regulating HBV associated with liver tumorigenesis [156] it was found the presence of HBx and MAT2A overexpression in most HCCs. In vitro experiments revealed that HBx activates MAT2A expression in HCC cells and this regulation requires the cis-regulatory elements of NF-kB and CREB on *MAT2A* gene promoter. HBx or MAT2A overexpression inhibits cell apoptosis. Furthermore, HBx induces MAT1A:MAT2A switch through NF-KB and CREB signaling pathways thus decreasing SAM production, inhibiting HCC cell apoptosis and enhancing HCC growth. HBx reduces MAT1A expression and SAM production and enhances MAT2β expression. Furthermore, SAM may inhibit HCV expression by modulating antioxidant enzymes, restoring the biosynthesis of GSH and switching MAT1A/MAT2A ratio in HCV expressing cells [157]. The addition of SAM to peginterferon and ribavirin improves the early viral kinetics and increases interferon-stimulated gene induction in patients with chronic HCV infection non-responders to previous therapy [152,158]. HCV protein also alters JAK-STAT signaling by inhibiting STAT1 methylation that favors STAT1 binding to its inhibitor PIAS1 [159]. SAM and betaine restore STAT1 methylation and increase the IFNalpha antiviral effect in cell culture [159]. Furthermore, among 29 patients with chronic hepatitis C, treated with the SAM, betaine, pegIFNα2b, and ribavirin, an early virological response occurred in 17 patients but only three patients achieved a sustained virological response to therapy [160]. A genome-wide association study [161] of virological response to a PEG-IFN-alpha/Ribavirin (RBV) combination therapy, in 293 Australians with genotype 1 chronic hepatitis C, showed the association of sustained virological response with the expression of the genomic region encoding IL28B (interleukin 28B). IL28B contributes to viral resistance and is upregulated by interferons and the RNA virus infection. These observations suggest the importance of the investigation of IL28B in the treatment of HCV. Moreover, a genetic polymorphism near the *IL28B* gene, in a region encoding interferon-lambda-3 (IFN-lambda-3), is associated with an approximately twofold change in response to treatment among U.S. patients of European ancestry [161]. Finally, SAM was found to improve the early virological response in chronic hepatitis C patients [158,160].

### 6.5. SAM ant Tumor Therapy

The term Cholangiocarcinoma represents a group of epithelial cancers with poor outcomes and different anatomical locations (intrahepatic, perihilar, and distal). Mixed hepatocellular cholangiocarcinomas represent a distinct subtype of primary liver cancers. Intrahepatic cholangiocarcinomas arise in cirrhotic liver [162]. The expression of MAfG (MAF bZIP transcription factor G) increases in cells and tissues with cholestasis, as well as in human cholangiocarcinoma and HCC in which MAFG overexpression correlates with tumor progression and reduced survival time [163,164]. c-Myc induction drives cholestatic liver injury and cholangiocarcinoma (CCA) in mice, and the induction of Maf proteins (MafG and c-Maf) contributes to cholestatic liver injury, whereas SAM administration could be protective [163]. MAT1A expression falls while MAfG and c-MAF expression increases in hepatocytes and bile duct epithelial cells during chronic cholestasis and in murine and human clear cell acanthoma [163]. The expression of MAfG increases in cells and tissues with cholestasis, as well as in human cholangiocarcinoma and HCC, and correlates with tumor progression and decree (sed survival time. SAM and UDCA reduce MAG expression, by distinct mechanisms.

OCA (obeticholic acid) induces MAfG expression, cancer cell proliferation, and growth of xenograft tumors in mice [164]. Furthermore, in 80 cholangiocarcinoma samples of patients treated with surgery, the overexpression of COX-2 and VEGF-C correlated positively with the clinical TNM stage but not with the differentiation status of tumor cells. The inhibition of COX-2 reduced VEGF-C mRNA expression and secretion of cholangiocarcinoma cells as well as their migration capacity, but not their proliferation. OCA is a synergistic agent in breast, and head and neck squamous cancer [165]. SAM inhibits VEGF-C expression [166]. These findings suggest the possibility that SAM alone or in association with other medicaments could contribute to cure cholangiocarcinomas. However, it must be noted that a recent analysis by Morgan et al. [167] aimed to determine if the oral SAM administration for 24 weeks of 2.4 g/d of SAM to 44 patients with hepatitis C cirrhosis and elevated α-fetoprotein would decrease serum α-fetoprotein (AFP) level, a biomarker of HCC risk, no difference in the α-fetoprotein content was found with respect to 43 patients treated with placebo. Changes in markers of liver function, liver injury, and hepatitis C viral level were not different between the two groups. Similarly, SAM did not change the markers of oxidative stress or serum GSH level. It must be considered that although the number of patient used in this study is limited, the negative results suggest that SAM cannot prevent the evolution of precancerous lesions to HCC at least in the patients with advanced precancerous lesions.

SAM, an antiapoptotic compound for normal hepatocytes, is proapoptotic for HCC [74,80]. It also induces apoptosis in MCF-7 breast cancer cells through the modulation of specific microRNAs [168], and in combination with Decitabine represses breast cancer growth and lung metastasis [169]. A synergistic antitumor effect of SAM plus Doxorubicin was also demonstrated in the hormone-dependent breast cancer cell lines [170]. The combination of SAM with Doxorubicin and Cisplatin induces apoptosis and cell migration in head and neck cancer cells [171]. Furthermore, since autophagy can act as an escape mechanism from the apoptotic activity of SAM in MCF-7 cells, the combination of SAM with chloroquine was proposed to kill these cells [172] miR-34a or miR-34c strengthens the pro-apoptotic effect of SAM, and activates p53 acetylation by inhibiting SIRT1 and HDAC1 expression [173].

SAM or methyl DNA-binding domain protein 2 antisense oligonucleotide (MBD2-AS) were found to inhibit the growth of the highly invasive human prostate cancer cells PC-3 [174]. This is associated with the inhibition of the expression of key genes, such as urokinase-type plasminogen activator (uPA), and matrix metalloproteinase-2 (MMP-2). as well as of the vascular endothelial growth factor expression and tumor cell invasion in vitro and in vivo in BALB/c nu/nu mice. SAM and MBD2 significantly decrease the methylation of the 5′ regulatory region and the expression of tumoral uPA and MMP-2 [174].

A study on the effect of SAM treatment on HT-29 and SW480 colorectal cancer cell lines [175], with distinct genetic features, showed that SAM reduces cell number by causing S phase arrest, and downregulated multiple genes related to epithelial-mesenchymal transition (e.g., TGFB1) in both cell lines. Remarkable increase of genomic stability was only observed in SW480 cells. Thus, SAM induced senescence, DNA repair, genome stability and reduced colorectal cancer cell progression. Furthermore, SAM or MTA treatment of colon cancer, induced in Balb/c mice by azoxymethane and dextran sulfate sodium, decreased tumor load by 40% and the expression of NF-KB, IL-6 and IL-10, STAR3, and AKT [82]. MTA, but not SAM, inhibited the expression of TNF-a and inducible iNOS. In vivo, both treatments induced apoptosis and inhibited the proliferation, beta-catenin, NF-kB, and interleukin 6 signaling [82].

SAM oncosuppressive action was also tested against the human osteosarcoma cells LM-7 and MG-63 that were treated with SAM or its inactive analog SAH as control [176]. SAM induced a dose-dependent inhibition of tumor cell proliferation, invasion, migration, and cell cycle. The inoculation of cells treated with SAM for six days into the tibia or via intravenous route in SCID (severe combined immune deficient) mice was followed by the development of skeletal lesions smaller and with marked reduction in pulmonary metastasis with respect to control groups. Different genes involved in osteosarcoma progression and signaling pathways implicated in bone formation, wound healing, and tumor progression were methylated in SAM-treated LM-7 cells.

### 6.6. SAM and Genetic Predisposition to Hepatocarcinogenesis

The research on the genetic backdrop of HCC in rodents demonstrated the existence of a polygenic predisposition, where the cancer phenotype was determined by the contribution of highly penetrant cancer-related genes and a complex system of epistatic interactions of various modifier genes [177]. A similar model applies to human hepatocarcinogenesis. Comparative functional genetics analysis identified the best-fit mouse [178] and rat [52,177] models of hepatocarcinogenesis.

**Figure 5:**
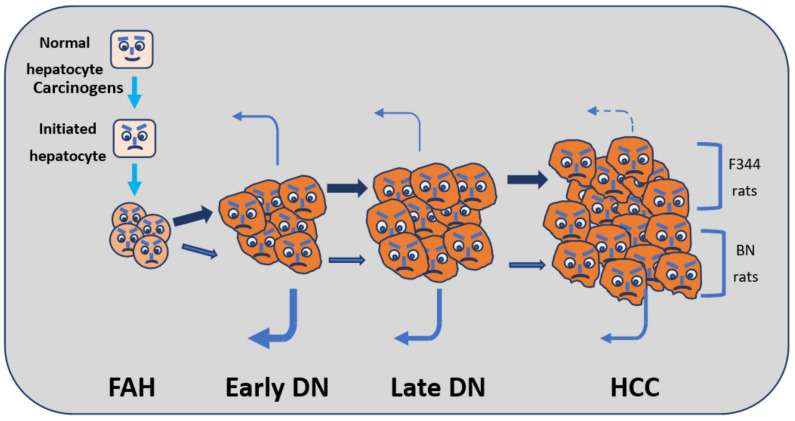
Multiphasic hepatocarcinogenesis. Retro-reverse arrows indicate remodeling. Arrows thickness is proportional to the rate and intensity of the changes. Abbreviations: DN, dysplastic nodules; FAH, foci of altered hepatocytes; HCC, hepatocellular carcinomas.

The induction of HCC in rats, according to the resistant hepatocyte protocol [2], is a multistep process in which cells initiated by chemical carcinogens form small aggregates of few cells that evolve to minifoci of 10–100 cells immunohistochemically positive to the placental isoform of glutathione-S-transferase (GST) (Figure 5). The proliferation of these cells leads to the formation of foci of altered hepatocytes (FAH), dysplastic nodules (DN), and HCCs (Figure 5). During this process, some cells re-differentiate (remodeling) [2]. Remodeling progressively decreases from foci to early nodules and to dysplastic nodules.

Previous work in our laboratory showed the rapid evolution of initiated cells to HCC in rats genetically susceptible to hepatocarcinogenesis, whereas in some resistant strains, the initiated cells evolve slowly, most preneoplastic lesions re-differentiate and only few HCCs appeared [52]. We found by a supervised hierarchical analysis of 6132 genes—common to rat and human liver—that DNs and HCCs of the resistant BN rats clustered with human HCC with better prognosis (HCCB), and most DNs and all HCCs of the susceptible F344 rats clustered with human HCC with poorer prognosis (HCCP) [52]. The differences between HCCB and HCCP were based on a higher size, Edmondson/Steiner grade, alpha-fetoprotein secretion, proliferation index, Midkine expression (as index of invasivity), and shorter patients’ survival in HCCP than in HCCB. These observations underlay the role of genetic predisposition on hepatocarcinogenesis, on HCC prognosis in mouse and rat models. Our supervised hierarchical analysis [52] indicates that genes implicated in hepatocarcinogenesis, such as *Anxa5*, c*-Myc*, *Ctgf*, and the IGF family, and genes *igfbp1* and *Igfbp3* are significantly more expressed in Nodules and HCCs of F344 susceptible rats and human HCCP, whereas *Bhmt*, implicated in the maintenance of a high SAM pool, *Dmbt1*, implicated in the malignant transformation of hepatic progenitor cells, and the ERK inhibitor *11* are more expressed in Nodules and HCCs of BN rats and human HCCP [52].

## 7. Conclusions

Two crucial discoveries motivated the research on SAM’s therapeutic role against liver preneoplastic and neoplastic lesions: the Mat1A/Mat2A switch with consequent decrease in SAM synthesis in HCC [15] and the decrease of SAM, associated with steatosis, in liver of ethanol-intoxicated rats [20] and in preneoplastic and neoplastic rat liver [17]. A strong evidence proved the beneficial effects of SAM in these conditions [20,21]. Some discrepancy, however, exists between the evidence of positive results of SAM treatment of experimental tumors and the more modest effects of SAM in the treatment of human disease. It must be considered, however, that the experimental curative effect of SAM is generally tested on relatively early stages of the development of experimental tumors, whereas for the therapy of human disease SAM is administered to more advanced and aggressive lesions. Indeed, SAM was found to be much more effectual when tested on HCCs less aggressive of BN rats and in human HCCBs.

Researches on the genetic background of liver cancer, in rodent models, proved the role of a polygenic predisposition to liver cancer, where highly penetrant cancer-related genes and a complex network of epistatic interactions of different modifier genes contribute to determine the cancer phenotype. Population research has shown that a similar model applies to human hepatocarcinogenesis [178]. Therefore, the detailed knowledge of the liver tumor epigenetics is fundamental for the diagnosis, prognosis, and therapy of this tumor. Comparative functional genetics studies identified the best-fit mouse and rat models of hepatocarcinogenesis and allowed the supervised hierarchical analysis of genes, common to mice, rat and human liver, involved in genetic predisposition to HCC. The identification of genes involved in the tumorigenesis of different tissues could be of capital importance for the prevention, early diagnosis, and therapy of tumors.

## Abbreviations

AFL: alcoholic fatty liver; AKT, serine/threonine kinase 1; ALD, alcoholic liver disease; LP, placental alkaline phosphatase; ALT, Alanine aminotransferase; AMPK, Akt-mediated adenosine monophosphate protein-activated kinase; AP-1, serine/threonine kinase 1; APEX1, apyrimidinic endodeoxyribonuclease 1; ASH, alcoholic steatohepatitis; AST, aspartate aminotransferase; ATP, adenosinetriphosphate; AUF1, AU-rich element RNA-binding factor 1; BHMT, betaine homocysteine methyltransferase; CYP2E1, cytochrome P4502E1; DN, dysplastic nodules; dSAM, decarboxylated SAM; ERK, extracellular signal-regulated kinase; ETOH. ethanol; FAH, foci of altered hepatocytes; FOXM1, forkhead box protein M1; GIT1, G-protein-coupled receptor interacting protein 1; GNMT, glycine N-methyltransferase; GSH, reduced glutathione; GST, glutathione-S-transferase; HCC, hepatocellular carcinoma; HCCB, hepatocellular carcinoma with better prognosis; HCCP, hepatocellular carcinoma with poorer prognosis; HCY, homocysteine; HGF, hepatocyte growth factor; HIF-, Hypoxia-inducible factor 1; Hur, human antigen R; HAUSP, herpesvirus-associated ubiquitin-specific protein; IHC, intrahepatic cholestasis; iNOS, inducible nitric oxide synthase; LKB1, Liver Kinase B1; MBD2-AS, methyl DNA-binding domain protein 2 antisense oligonucleotide; MAFG, MAF bZIP transcription factor G; MAT, methionine adenosyltransferase; MeTHF, 10-methylenetetrahydrofolate;mMeTHFR, 5-10,methyltetrahydrofolate reductase; MTA, 5′-methylthioadenosine; MTHF, 5-methyltetrahydrofolate; MeTHFR, 5-10,methyltetrahydrofolate reductase; mTORC2, mammalian target of rapamycin complex; NAFLD, non-alcoholic fatty liver disease; NASH, non-alcoholic steatohepatitis; NF-kB, nuclear factor kappa B; ODC, ornithine decarboxylase; PEMT, phosphatidylethanolamine methyltransferase; PP2A, Protein phosphatase 2A;ROS, reactive oxygen species; SAH, S-adenosylhomocysteine; SAM, S-adenosylmethionine;mSFRT-1; sirtuin-1; THF, 5-tetrahydrofolate; TNF, Tumor Necrosis Factor; UDCA, ursodeoxycholic acid; uPA, urokinase-type plasminogen activator; VEFG-C, vascular endothelial growth factor-C.
